# Norovirus Epidemiology and Genotype Circulation during the COVID-19 Pandemic in Brazil, 2019–2022

**DOI:** 10.3390/pathogens13010003

**Published:** 2023-12-19

**Authors:** Sylvia Kahwage Sarmento, Juliana da Silva Ribeiro de Andrade, Fábio Correia Malta, Alexandre Madi Fialho, Mateus de Souza Mello, Fernanda Marcicano Burlandy, Tulio Machado Fumian

**Affiliations:** Laboratory of Comparative and Environmental Virology, Oswaldo Cruz Institute, Oswaldo Cruz Foundation, Rio de Janeiro 21045-900, RJ, Brazilfburlandy@ioc.fiocruz.br (F.M.B.)

**Keywords:** norovirus, molecular epidemiology, viral load, RT-qPCR, genotyping, Brazil

## Abstract

Norovirus stands out as a leading cause of acute gastroenteritis (AGE) worldwide, affecting all age groups. In the present study, we investigated fecal samples from medically attended AGE patients received from nine Brazilian states, from 2019 to 2022, including the COVID-19 pandemic period. Norovirus GI and GII were detected and quantified using RT-qPCR, and norovirus-positive samples underwent genotyping through sequencing the ORF1/2 junction region. During the four-year period, norovirus prevalence was 37.2%, varying from 20.1% in 2020 to 55.4% in 2021. GII genotypes dominated, being detected in 92.9% of samples. GII-infected patients had significantly higher viral concentrations compared to GI-infected patients (median of 3.8 × 10^7^ GC/g and 6.7 × 10^5^ GC/g, respectively); and patients aged >12–24 months showed a higher median viral load (8 × 10^7^ GC/g) compared to other age groups. Norovirus sequencing revealed 20 genotypes by phylogenetic analysis of RdRp and VP1 partial regions. GII.4 Sydney[P16] was the dominant genotype (57.3%), especially in 2019 and 2021, followed by GII.2[P16] (14.8%) and GII.6[P7] (6.3%). The intergenogroup recombinant genotype, GIX.1[GII.P15], was detected in five samples. Our study is the first to explore norovirus epidemiology and genotype distribution in Brazil during COVID-19, and contributes to understanding the epidemiological dynamics of norovirus and highlighting the importance of continuing to follow norovirus surveillance programs in Brazil.

## 1. Introduction

Noroviruses are recognized as the leading cause of acute gastroenteritis (AGE) in people of all ages worldwide, causing around 20% of AGE episodes and over 200,000 deaths annually in low- and middle-income countries [1,2,3,4]. In countries where the rotavirus vaccine has been implemented, cases of norovirus-related illnesses frequently rank as the primary reason for medical visits due to AGE in pediatric populations [5,6,7].

Noroviruses are members of the *Norovirus* genus within the *Caliciviridae* family. They are positive-sense, single-stranded RNA viruses, with an approximately 7.5 kb genome that contains three open reading frames (ORFs 1–3). ORF1 encodes nonstructural proteins, including RNA-dependent RNA polymerase (RdRp), while ORF2 and ORF3 encode the major capsid protein (VP1) and the minor capsid protein (VP2), respectively [8]. Recombination between the overlap of ORF1 and ORF2 frequently occurs, leading to the appearance of novel strains containing different RdRp and VP1 combinations [9]. Based on the VP1 amino acid sequences, noroviruses are classified into ten different genogroups (GI-GX) and divided into 48 genotypes, but only GI, GII, GIV, GVIII and GIX are known to infect humans [10,11]. GI and GII norovirus are responsible for the majority of disease in humans and notably, the GII.4 has been the dominant genotype, responsible for the majority of norovirus-associated outbreaks over the past two decades [12]. Since the mid-1990s, new pandemic GII.4 variants have emerged approximately every three to five years, and have caused large outbreaks worldwide [13,14]. The emergent variant replaces previous circulating GII.4 strains due to amino acid substitutions on antigenic sites/motifs (A-I) located at the outermost surface of the VP1, which allows the new virus to escape previously acquired immunity [15,16]. Other GII.4 variants have been detected with limited geographical distribution (e.g., the recently emergent GII.4 Hong Kong variant, reported in Asia and Europe between 2017 and 2019 [17].

Although the GII.4 strains are the most common strains detected among all age groups, non-GII.4 strains, such as GII.2, GII.17, and GII.6 viruses, are common causes of sporadic cases and illness in young children [18,19]. At least two norovirus vaccines are in phase III clinical trials, and others are in phases I and II, and developmental stages (https://clinicaltrials.gov/study/NCT05916326?cond=norovirus&page=3&rank=25, accessed on 23 October 2023) [20,21]. However, their design is challenging because of the high genetic diversity of noroviruses and incomplete understanding of cross-protective immunity [22].

Global surveillance of norovirus faces notable challenges, which hinder the precise assessment of its global disease burden. Estimates of norovirus burden and genotype circulation, especially in low- and middle-income countries, are limited and significantly underreported. In Brazil, different epidemiological patterns and a large genetic diversity of norovirus have been found in recent years [23,24,25]. A previous study from our group, carried out before the COVID-19 pandemic, has shown the high incidence (32.1%) of norovirus in AGE cases in Brazil, and its high genetic diversity over the period [26]. Globally, few studies have described norovirus epidemiology and genotype circulation during the COVID-19 pandemic [27,28,29,30,31]. Therefore, there is a gap of information concerning the interplay between COVID-19 interventions and the epidemiology of norovirus.

In Brazil, the impact of implementing extensive non-pharmaceutical interventions to combat SARS-CoV-2 on the dynamics of norovirus circulation is still unknown. This includes examining potential shifts in the disease’s behavior, seasonality, burden across different age groups, and the circulating genotypes. In the present study, we aimed to continue investigate norovirus prevalence and genotype circulation in AGE cases over a four-year period (2019–2022) in Brazil. Stool samples from medically attended AGE patients were tested for norovirus GI and GII by RT-qPCR, and viral loads were estimated in order to evaluate differences in RNA shedding among different genogroups and age groups. Norovirus genotypes were determined by sequencing the ORF1-2 genomic region. This is the first report on the epidemiological characteristics of norovirus infections and their genetic diversity within the context of the COVID-19 pandemic in Brazil.

## 2. Materials and Methods

### 2.1. Stool Samples

This study included stool samples that were collected between January 2019 and December 2022 from outpatients (children and adults) with symptoms of AGE. For instance, AGE was characterized as ≥three liquid/semi liquid evacuations in a 24 h period. Stool samples were collected from medically attended AGE patients from ten states within three regions of Brazil and were systematically sent together with clinical-epidemiological records to the Laboratory of Comparative and Environmental Virology that houses the Regional Rotavirus Reference Laboratory (RRRL) at the Oswaldo Cruz Institute. The RRRL is part of the national rotavirus surveillance program, overseen by the General Coordination of Public Health Laboratories within the Brazilian Ministry of Health (MoH). In addition to rotavirus surveillance, the RRRL performs norovirus diagnostic tests for all received stool samples.

### 2.2. Ethics Statements

This study was approved by the Oswaldo Cruz Foundation (Fiocruz) Ethics Committee (approval number CAAE: 94144918.3.0000.5248). The surveillance was performed through a hierarchical network in which samples are provided by medical requests in hospitals and health centers that are monitored by the Brazilian Unified Health System (SUS). This study was conducted within the scope of the RRRL/MoH as part of a Federal public health policy for viral AGE surveillance in Brazil. Patient-informed consent was waived by the Fiocruz Ethical Committee, and patients’ data were maintained anonymously and securely.

### 2.3. Viral RNA Extraction

Viral nucleic acids were purified from 140 μL of clarified stool suspension (10% *w*/*v*) prepared with Tris-calcium buffer (pH = 7.2). Samples were subjected to an automatic nucleic acid extraction procedure using the QIAcube^®^ automated system and QIAamp^®^ Viral RNA Mini kit (both from QIAGEN, Hilden, Germany), according to the manufacturer’s instructions. Nucleic acids were eluted in 60 µL of the elution buffer AVE. The isolated DNA/RNA was immediately stored at −80 °C until the molecular analysis. In each extraction procedure, RNAse/DNAse-free water was used as negative control.

### 2.4. Norovirus Detection and Quantification

Norovirus GI and GII were detected and quantified by using a TaqMan-based quantitative one step RT-PCR (RT-qPCR) duplex protocol with primers and probes targeting the ORF1/2 junction region [32]. Briefly, RT-qPCR reactions were performed with 5 µL of the extracted RNA in a final volume of 25 µL using the SuperScript™ III Platinum™ One-Step RT-qPCR Kit (ThermoFisher Scientific, Invitrogen Division, Carlsbad, CA, USA) in the Applied Biosystems^®^ 7500 Real-Time PCR System (Applied Biosystems, Foster City, CA, USA). The thermal cycling conditions were carried out as follows: RT step at 50 °C for 60 min, an initial denaturation step at 95 °C for 5 min and 40 cycles of PCR amplification at 95 °C for 15 s and 60 °C for 1 min. All samples that crossed the threshold line showing a characteristic sigmoid curve with a cycle threshold (Ct) value < 35 were regarded as positive. All runs included negative and non-template controls (NTC) to ensure the correct interpretation of the results throughout the study. To estimate norovirus viral load, a standard curve prepared by six 10-fold serial dilutions (10^6^–10^1^ genome copies (GC) per reaction) of a double-stranded DNA fragment (gBlock^®^ GeneFragment, Integrated DNA Technologies, Coralville, IA, USA) containing the norovirus amplification region sequence was used in each RT-qPCR reaction. Norovirus viral loads were expressed as genome copies per gram (GC/g) of stool.

### 2.5. Molecular Characterization and Genotyping

Norovirus-positive samples obtained by RT-qPCR were subjected to conventional one-step RT-PCR for dual genotyping of polymerase and capsid regions. The reactions were performed using the Qiagen One Step RT-PCR kit (QIAGEN, Hilden, Germany) with primers Mon 432/G1SKR for GI and Mon 431/G2SKR for GII, that amplifies the ORF1/2 junction region [33,34]. The generated amplicons fragments [543 and 557 base pairs (bp) for GI and GII, respectively were purified using the ExoSAP clean-up kit (ThermoFisher Scientific, Waltham, MA, USA) or the QIAquick PCR Purification Kit (QIAGEN, Hilden, Germany). Purified DNA were Sanger sequenced in both directions at the FIOCRUZ Institutional Platform for DNA sequencing (PDTIS) on an ABI Prism 3730*xl* genetic analyzer (Applied Biosystems, Waltham, MA, USA).

Norovirus consensus sequences were obtained after nucleotide (nt) alignment and editing using Geneious prime 2021.2.2 (Biomatters Ltd., Auckland, New Zealand), and genotypes were confirmed in terms of closest homology sequence, using Basic Local Alignment Search Tool (BLAST v. 2.15.0). Norovirus genotypes were firstly assigned based on the new nomenclature system using the two norovirus typing tools https://www.rivm.nl/mpf/typingtool/norovirus and https://nrovirus.ng.philab.cdc.gov (accessed on 10 October 2023).

### 2.6. Phylogenetic Analysis

Phylogenetic trees were constructed using the neighbor-joining method for each ORF of GI and GII. The best substitution models were selected based on the corrected Akaike information criterion (AICc) value as implemented in MEGA X v. 10.1.7 [35]. The model used in this study was Kimura 2-parameter (K2) + G (ORF1 and ORF2) (2000 bootstrap replications for branch support). Norovirus reference sequences were obtained from the National Center for Biotechnology Information (NCBI) database. Nucleotide sequences obtained in this study were submitted to NCBI GenBank (accession numbers: OR780022–OR780025; OR780639–OR780641; OR780757–OR780760; OR780762–OR780775; OR780777; OR780778; OR791432–OR791498; OR791502; OR791503; OR791584–OR791594; OR791596–OR791599; OR791660; OR791689; OR791721–OR791739; OR791748–OR791769; OR791787–OR791810; OR791846–OR791863; OR791939–OR792118; OR792344; OR792345).

### 2.7. Statistical Analysis

Statistical analyses were performed using GraphPad Prism v. 8.4.1 (GraphPad Software, San Diego, CA, USA). Box-and-whisker plots were produced to illustrate differences between medians within interquartile ranges. The Mann–Whitney U test was used for comparison of viral load values between genogroups and age groups. The Chi-square and Fisher’s exact tests were used for analyzing categorical characteristics in contingency tables. For all analyses, a *p*-value < 0.05 was considered statistically significant.

## 3. Results

### 3.1. Norovirus Epidemiology

During the four-year study period (2019–2022), we tested a total of 2913 stool samples from medically attended patients with AGE. Overall, we detected norovirus in 37.2% of samples (n = 1085), with 33.8% (248/732) in 2019, 20.1% (61/302) in 2020, 55.4% (574/1035) in 2021, and 23.9% (202/844) in 2022. A significantly higher detection rate was observed in 2021 compared to other years (*p* < 0.005). Norovirus GII was the predominant genogroup, being detected in 92.9% (1009/1085) of samples, followed by GI in 6% of samples (65/1085), and co-detection of GI and GII was observed in 1.1% of samples (n = 11). Regarding regional analysis, the norovirus detection rate was significantly higher in the Southern region (41.9%) of Brazil compared to the Southeastern (27.4%, *p* < 0.0001) and Northeastern (34.1%, *p* = 0.0005) regions. Additionally, over the period analyzed, two states from south Brazil (Santa Catarina and Rio Grande do Sul) accounted for more than 65% of all norovirus-positive samples (n = 734). Table 1 shows a detailed analysis of norovirus detection by regions and states.

**Figure 1:**
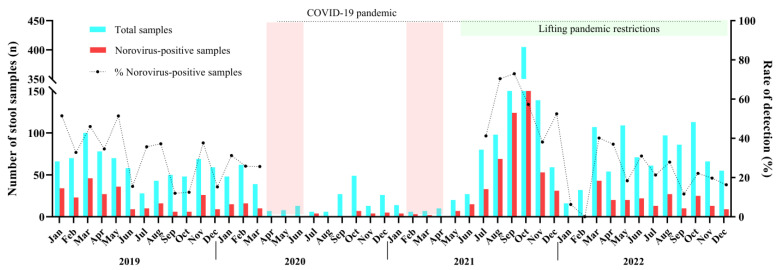
Monthly distribution of tested acute gastroenteritis samples (AGE), norovirus-positive samples and norovirus detection rates in Brazil during 2019–2022. The dotted line was the period where non-pharmaceutical interventions (wearing masks, surface disinfection, social distancing, hand hygiene, and school closure) were adopted during COVID-19 pandemic. The two pink columns show the strict lockdown period in Brazil.

We detected norovirus year-round without a marked seasonality. During 2019 up to March 2020, norovirus circulated at mean rate of 35%, varying from 12% in September/2019 to 51.5% in January/2019. Between April 2020 and June 2021, a smaller number of AGE cases (8.2%, n = 239/2913) were reported compared to the other months of this study. The low levels of norovirus infections (n = 54) and AGE cases observed for one year and 2 months coincides with the period when intervention measures, such as social distancing and school closures, were implemented in Brazil to control the spread of SARS-CoV-2 during the COVID-19 pandemic. Afterwards, during the second half of 2021, norovirus infections peaked in Brazil with detection rates varying from 70.4% in August to 72.9% in September, especially in some states. Several cities of the state of Rio Grande do Sul recorded norovirus-associated outbreaks, which led to an increased number of tested and positive samples during the period. In 2022, after the peak in the second semester of 2021, norovirus prevalence decreased, and the annual positivity mean rate recorded was 23.9% (Figure 1).

Concerning seasonal distribution, the norovirus detection rates were higher in winter (40%) and spring (41.4%) months than in summer and autumn months. During spring months, the difference was statistically significant compared to the summer (29.1%, *p* < 0.0001) and autumn (32.9%, *p* = 0.0032) months (Table 2).

Regarding age groups, norovirus detection rates varied from 26.4% (64/242) in the group of children between 0 and 6 months old to 48.5% (312/642) in children aged between >12–24 months old. The high detection rates observed were among children between 6 and 24 months old. The norovirus detection rate was significantly higher among children in the age group of >12–24 months old compared to other age groups. The majority of norovirus-positive samples in our study were from children less than five years old, representing 68.2% (740/1085). Regarding patients within the >60 months group, comprised of older children (>5–10 y) adolescents (>10–20 y), adults (>20–60 y), and elders (≥60 y), norovirus was detected in 28.7% (46/160), 26.2% (41/156), 33.1% (189/570), and 37.6% (70/186) of samples, respectively (Table 3). As for gender, norovirus was detected in 48.7% and 51.2% of samples from males and females, respectively, without statistical significance (*p* = 0.9960).

**Figure 2:**
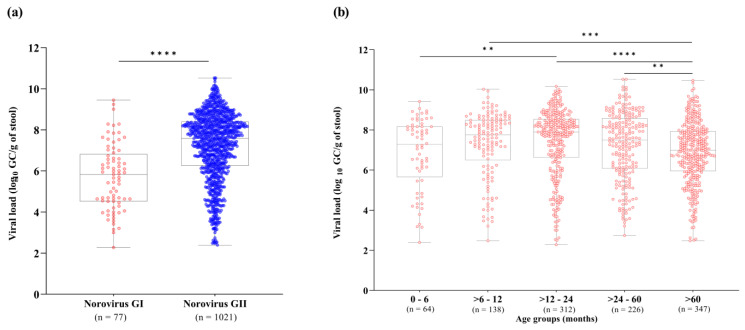
Norovirus viral load expressed as log_10_ genome copies per gram of stool (GC/g) among genogroups I (GI) and GII (**a**), and different age groups (**b**) detected in Brazil, from 2019 to 2022. Box-and-whisker plots show the first and third quartiles (equivalent to the 5th and 95th percentiles), the median (the horizontal line in the box), and range of norovirus viral load concentrations. **** *p*  ≤  0.0001, *** *p*  ≤  0.001, ** *p*  ≤  0.01.

We also investigated norovirus stool shedding among different norovirus genogroups and age groups. RT-qPCR results demonstrated that norovirus GII-infected patients showed significantly higher viral loads in stool compared to GI-infected patients (*p* < 0.0001). Viral loads of norovirus GI and GII ranged from 1.8 × 10^2^ to 2.8 × 10^9^ GC/g of stool (median of 6.7 × 10^5^ GC/g), and 2.4 × 10^2^ to 3.3 × 10^10^ GC/g of stool (median of 3.8 × 10^7^ GC/g), respectively (Figure 2a). Concerning age groups, norovirus viral loads varied broadly among patients. We observed significantly lower norovirus viral loads in patients over sixty months old (median value of 9.9 × 10^6^ GC/g, n = 347) compared to the age group of >6–12 months old (median of 5.7 × 10^7^ GC/g), >12–24 months old (median of 8 × 10^7^ GC/g) and >24–60 months old (median of 3.1 × 10^7^ GC/g). Higher stool shedding was observed in norovirus-infected children aged between 6 and 24 months old. Among all age groups, the highest median viral load (8 × 10^7^ GC/g) was observed in children aged >12–24 months old (Figure 2b).

### 3.2. Norovirus Genotyping

**Figure 3:**
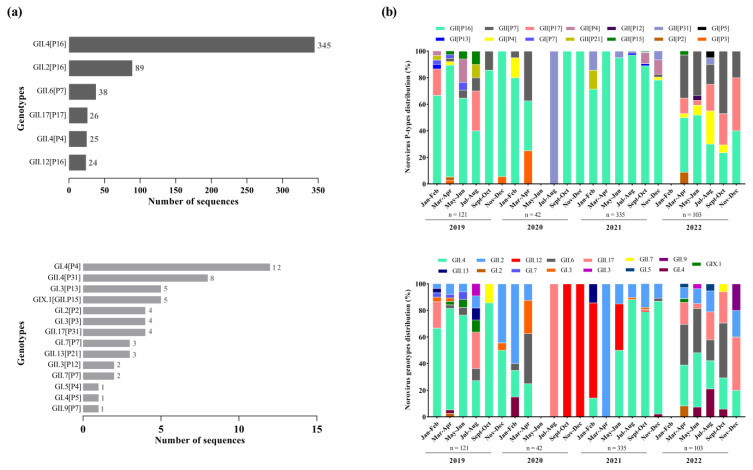
Distribution of norovirus genotypes identified with high and low frequencies from genotyped samples in Brazil between 2019 and 2022. (**a**) Bimonthly distribution of norovirus P-types and capsid genotypes identified during the study period. (**b**) Numbers to the right of the bars indicate the number of sequences detected for each dual type.

We successfully sequenced 55.4% of norovirus-positive samples (602/1085), with 48.8% (121/248), 68.8% (42/61), 58.3% (335/574) and 51.4% (104/202) being from 2019, 2020, 2021, and 2022, respectively. The dual genotyping of polymerase and capsid regions showed the circulation of different norovirus genotypes (n = 20), however, GII.4 Sydney[P16] was by far the dominant genotype (57.3%; n = 345) during the period, especially in 2019 and 2021, when this genotype was detected in 62.8% and 65.5%, respectively, among the sequenced strains. The second most common genotype was GII.2[P16] (14.8%; n = 89), followed by GII.6[P7] (6.3%; n = 38), GII.17[P17] (4.3%; n = 26), GII.4[P4] (4.1%; n = 25), and GII.12[P16] (3.9%; n = 24) (Figure 3a). Besides those common genotypes, we also detected, less frequently, 14 other genotypes. Thirteen recombinant norovirus genotypes were detected, with the majority of the recombinant strains belonging to GII (n = 9), including GII.12[P16], GII.13[P21], GII.17[P31], GII.2[P16], GII.3[P12], GII.4[P16], GII.4[P31], GII.6[P7], and GII.9[P7] (Figure 3a).

Overall, we found a greater diversity of polymerase types and capsid genotypes of norovirus GI and GII over the period. Concerning the RdRp region (Figure 3b), we characterized six types of norovirus GI (GI.P2, GI.P3, GI.P4, GI.P5, GI.P7 and GI.P13) and eight types of norovirus GII (GII.P4, GII.P7, GII.P12, GII.P15, GII.P16, GII.P17, GII.P21 and GII.P31). The GII.P16 was the predominant P-type, especially in the first three years of the study (2019–2021) when this polymerase type was detected at rates over 60%, and in combination with different genotypes (especially GII.4, GII.2, and GII.12). During 2022, we observed the emergence of other genotypes with different P-types, especially GII.P17 and GII.P7, which co-circulated at similar rates to GII.P16 (Figure 3b).

Based on the VP1 characterization, we identified five genotypes of GI (GI.2, GI.3, GI.4, GI.5 and GI.7), nine genotypes of norovirus GII (GII.2, GII.3, GII.4, GII.6, GII.7, GII.9, GII.12, GII.13 and GII.17), and one GIX genotype (GIX.1). The GII.4 was the most prevalent genotype mainly during 2019 and 2021, being detected in combination with different P-types (GII.P4, GII.P16 and GII.P31). The GII.2, that was the second most common genotype, circulated in combination with a single P-type (GII.P16). During the second half of 2020, only two genotypes were detected: GII.17 in July and August, and GII.12 between September and December. In 2022, in contrast to the other years, we observed a similar proportion of different genotypes circulating throughout the year, without the dominance of a specific genotype. Several polymerase types were associated with more than one capsid genotypes, such as GII.P16 (GII.4[P16] and GII.2[P16]), GII.P7 (GII.6[P7], GII.7[P7], and GII.9[P7]), and GII.P31 (GII.4[P31] and GII.17[P31]).We also detected one intergenogroup recombinant genotype (GIX.1[GII.P15]) in five samples from the states of Bahia, Rio de Janeiro, and Santa Catarina (Figure 3b).

**Figure 4:**
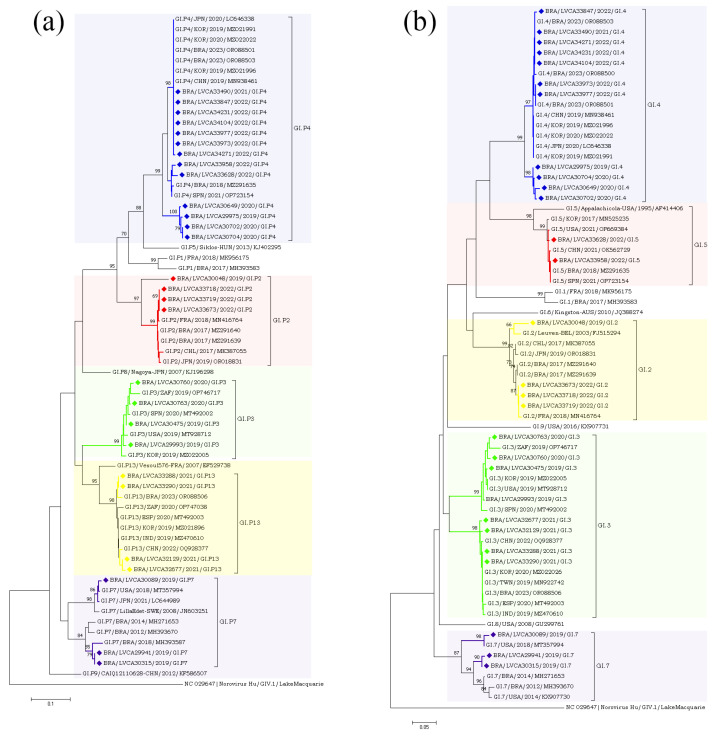
Phylogenetic analyses based on ORF1 (**a**) and ORF2 (**b**) nucleotide (nt) sequences of GI norovirus Brazilian strains. Reference strains were downloaded from GenBank and labelled with their genotypes followed by country, year, and accession number. Strains obtained (marked with a diamond) are shown as per country followed by the LVCA, internal register number, year, and genotype of collection (i.e., BRA/LVCA33973/2022/GI.P4). The neighbor-joining phylogenetic tree was constructed with MEGA X software v. 10.1.7 and bootstrap tests (2000 replicates), based on the Kimura two-parameter model. Bootstrap values above 60% are given at branch nodes.

The partial nucleotide sequences of the RdRp and capsid genes of the norovirus GI (n = 30) and GII (n = 572) strains detected in our study were used for phylogenetic analysis, together with the reference strains. Regarding norovirus GI, the most frequent genotype was GI.4[P4], detected in 40% of the samples (n = 12), followed by GI.3[P13] in 16.6% (n = 5) (Figure 4). The genotypes of GI.2[P2], GI.3[P3], GI.4[P5], GI.7[P7], and GI.5[P4] were identified in less than 15% of the genotyped samples. Interestingly, GI.P4 sequences that were associated with the GI.4 and GI.5 capsid genotypes grouped into two different clusters with previous strains from Brazil (GI.P4/BRA/2023/OR088503 and GI.P4/BRA/2018/MZ291635) detected in 2018 and 2023. Despite GI.P7 sequences being associated with a single capsid genotype (GI.7), they grouped into two different clusters with strains from the USA (GI.P7/USA/2018/MT357994) and Brazil (GI.P7/BRA/2018/MH393587), both isolated in 2018. Brazilian sequences GI.P2 and GI.P13 showed a high similarity (>98% nt sequence identity) with the Brazilian strains detected in 2017 and 2023, respectively (Figure 4a). Additionally, four GI.4 strains, isolated in 2020 and 2019, showed a high similarity to each other, with 98% of nucleotide sequence identity, and with Brazilian sequences grouped into two genetic clusters. Seven GI.4 strains that was isolated in 2021 and 2022 were closely related (>97% of nt identity) to previous strains from Brazil (GI.4/BRA/2023/OR088503, OR088501, and OR088500) detected in 2023. The GI.2 sequences showed a higher similarity with strains previously detected in France (GI.2/FRA/2018/MN416764) and Belgium (GI.2/BEL/2003/FJ515294). Brazilian GI.3 sequences that were associated with two different polymerase types (GI.P3 and GI.P13) grouped into two different clusters (>98% of nt identity) (Figure 4b).

**Figure 5:**
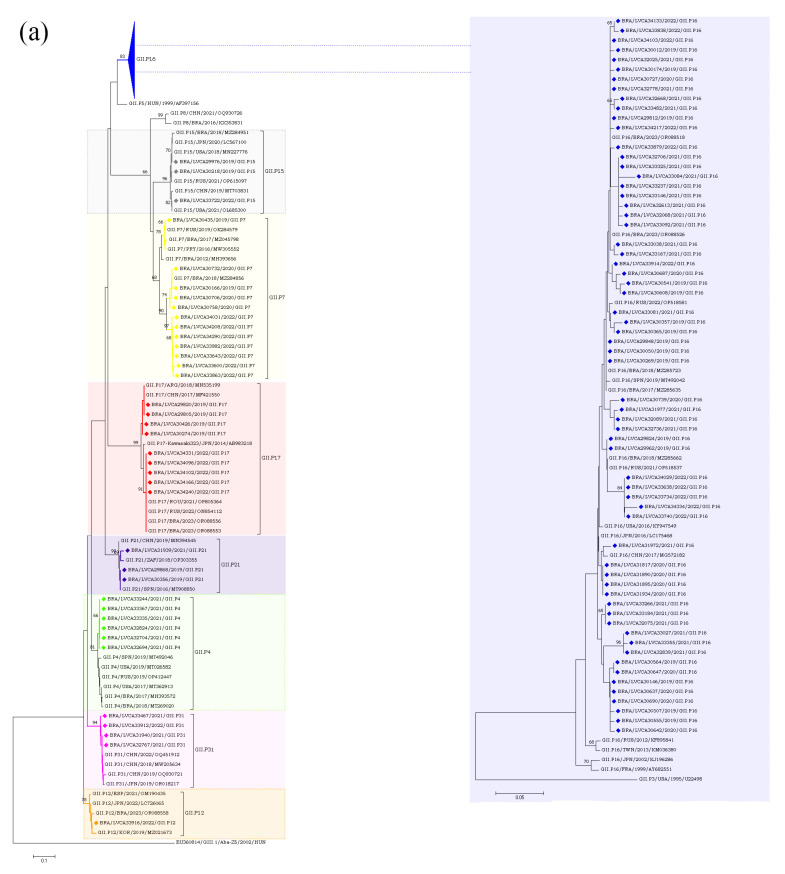

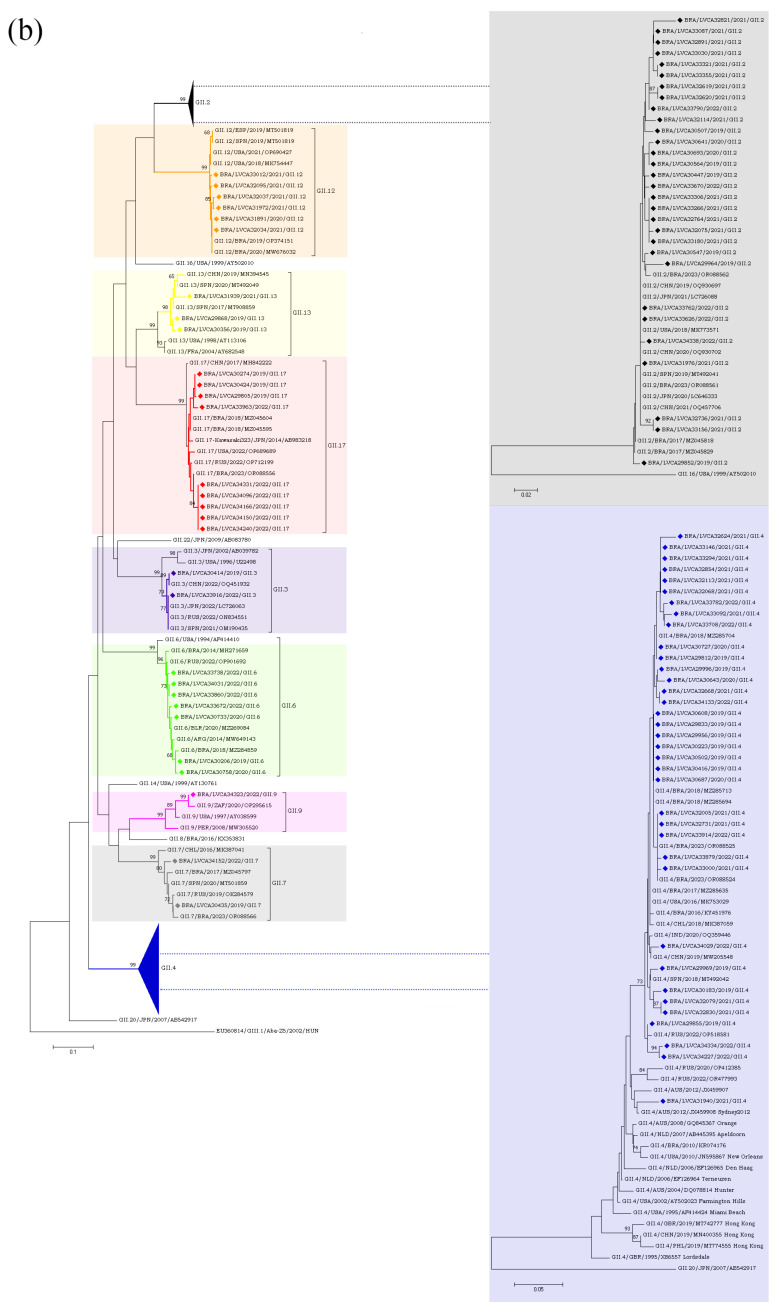
(**a**). Phylogenetic analyses based on ORF1 nucleotide (nt) sequences of GII norovirus Brazilian strains. Reference strains were downloaded from GenBank and labelled with their genotypes followed by country, year, and accession number. Strains obtained (marked with a diamond) are shown as per country followed by the LVCA, internal register number, year, and genotype of collection (i.e., BRA/LVCA30732/2020/GII.P7). The neighbor-joining phylogenetic tree was constructed with MEGA X software v.10.1.7 and bootstrap tests (2000 replicates), based on the Kimura two-parameter model. Bootstrap values above 60% are given at branch nodes. (**b**). Phylogenetic analyses based on ORF2 nucleotide (nt) sequences of GII norovirus Brazilian strains. Reference strains were downloaded from GenBank and labelled with their genotypes followed by country, year, and accession number. Strains obtained (marked with a diamond) are shown as per country followed by the LVCA, internal register number, year, and genotype of collection (i.e., BRA/LVCA34323/2022/GII.9). The neighbor-joining phylogenetic tree was constructed with MEGA X software v.10.1.7 and bootstrap tests (2000 replicates), based on the Kimura two-parameter model. Bootstrap values above 60% are given at branch nodes.

Regarding the GII genotypes, the GII.P16 polymerase type was found in cases involving GII.2, GII.12, and GII.4, whilst GII.P31 was associated with GII.4 and GII.17. Type GII.P7 was detected in association with three genotypes (GII.6, GII.7 and GII.9) and segregated into three different clusters. Brazilian GII.P17 strains detected in 2019 clustered with strains detected in Argentina (GII.P17/ARG/2018/MN535199) and China (GII.P17/CHN/2017/MF421550), whilst the five GII.P17 strains detected in 2022 were more closely related to strains previously detected in Brazil (GII.P17/BRA/2023/OR088556, OR088553), Russia (GII.P17/RUS/2022/ON854112), and Romania (GII.P17/ROU/2021/OP805364). The GII.2 sequences (n = 30) clustered with strains from Brazil (GII.2/BRA/2017/MZ045818;MZ045829 and GII.2/BRA/20123/OR088562), China (GII.2/CHN/2019/OQ930697 and GII.2/CHN/2020/OQ930702), Japan (GII.2/JPN/2020/LC646333 and GII.2/JPN/2021/LC726088), Spain (GII.2/SPN/ 2019/MT492041), and the USA (GII.2/USA/2018/MK773571) (Figure 5a,b).

## 4. Discussion

Our study provides information regarding the prevalence and the virological and molecular epidemiological features of norovirus among the AGE cases from medically attended patients collected in ten Brazilian states during the COVID-19 pandemic. Overall, we detected norovirus in 37.2% of samples from medically attended patients with AGE collected during 2019 and 2022. The norovirus GII genotype was more prevalent compared to GI among all age groups, and accounted for more than 90% of total infections. We identified several different genotypes circulating during the period, with the major dominance of the GII.4[P16] and GII.2[P16] viruses, followed by four additional GII genotypes. This is the first report of norovirus molecular epidemiology in Brazil during the course of the COVID-19 pandemic.

The percentage of norovirus infections in this study was similar to a previous study from our group that reported a norovirus prevalence of 32.1% from symptomatic outpatients during a two-year period (2017–2018), with monthly detection rates varying from 11% to 61% [26]. In Brazil, previous studies performed in other regions (Northern and Northeastern regions) have demonstrated norovirus detection rates varying from 11% to 35.2% in medically attended and hospitalized children with AGE [36,37,38]. In our study, as expected, the GII genotypes predominated among the infections, in line with several studies performed elsewhere, where norovirus GII detection rates varied from 70% to 93.6% among AGE cases [39,40,41]. During norovirus outbreaks reported in Catalonia, Spain from 2017 to 2019 in closed and semi-closed settings, GII was the predominant genogroup, being involved in 70% of outbreaks [31]. Arowolo et al. [42] showed that all norovirus positive samples belonged to GII in a recent study with children below five years old with AGE in Southwest Nigeria. Regarding Latin American countries, our findings are consistent with a study carried out in Argentina that detected norovirus GII in 88.8% of AGE outbreaks from 2013 to 2018 [19]. Lucero et al. [43] also reported a highest detection rate of norovirus GII (85%) in norovirus infections in Chile. A recent study found all norovirus infections in patients with acute gastroenteritis in rural and low-income urban areas in northern Brazil, from 2010–2016, belonged to GII [44].

Brazil’s first COVID-19 case and fatality were confirmed on 25 February and 17 March 2020, respectively. Subsequently, from April onward, a rapid surge in both COVID-19-confirmed cases and fatalities was observed. In response to the escalating situation, Brazilian Federal and State governments implemented a series of measures to control the spread of SARS-CoV-2. These initiatives encompassed the enforcement of social distancing protocols and lockdowns in severely affected regions, childcare and school closures, face masking, and, when available, widespread free testing demonstrating a concerted effort to mitigate the impact of the pandemic on public health. Especially when those measures were stringent, between April 2020 and June 2021, we observed a sharp decrease in the number of reported AGE and norovirus infections in Brazil. Similarly, several countries experienced a notable reduction in the circulation of norovirus during the pandemic, attributing the decline to enhanced hygiene practices, stringent public health measures, and heightened awareness of preventive measures, collectively contributing to a diminished prevalence of norovirus infections. Many studies have reported the decline in the incidence of several pediatric infectious diseases of viral etiology, including gastroenteric viruses [45,46,47,48]. For instance, in Victoria, Australia, the incidence of enteroviruses and norovirus, transmitted primarily through the fecal-oral route, declined significantly in 2020 compared to the previous decade [49]. In England, Douglas et al. [50] reported a substantial and sustained reduction in norovirus outbreaks during 2020 after SARS-CoV-2 control measures were implemented. A multicenter surveillance study conducted with hospitalized pediatric patients in Japan observed a marked reduction in enteric virus detection, with a 27.8% reduction in patients with norovirus post-COVID-19 pandemic [51]. In Brazil, we observed a surge of norovirus cases from July 2021, coinciding with restrictions being lifted countrywide. In the same line, a study in Shanghai, China, showed that the detection rate of norovirus among children ≤5 years of age with AGE symptoms abruptly decreased in 2020, the first year of the COVID-19 pandemic, compared to 2021 and 2022 [27]. Mathematical modeling studies have suggested that, if contact patterns return to pre-pandemic levels, the population’s susceptibility to rotavirus and norovirus infections would likely increase [52,53].

Concerning norovirus shedding, GII-infected patients showed a significantly higher viral load in stool (median of 3.8 × 10^7^ GC/g) compared to GI-infected patients (median of 6.7 × 10^5^ GC/g), similar to previous findings found by our group [26]. Shioda et al. [54] found that GII-positive specimens had slightly lower Ct values compared with GI-positive samples in sporadic cases, outbreaks, and asymptomatic controls in the United States and Latin America. Evidence of the association between viral load and genogroup is limited, but some data indicate that shedding may be higher for the GII genotypes due a better affinity to binding with cellular receptors, for instance HBGAs [55,56]. Moreover, a significantly longer viral shedding period could be associated with gene mutations or the recombination of the norovirus [57]. Cheng et al. observed in a study with hospitalized pediatric patients with confirmed norovirus gastroenteritis from 2015 to 2018, that the average viral load was highest in GII.4 Sydney infections, followed by GII.2[P16] infections and lowest in non-GII.4 infections [58]. In another study carried out in inpatients in Hong Kong, China, the viral load of recombinant genotype GII.2[P16] was higher than other genotypes in different age groups [59].

As for seasonal distribution, our study demonstrated that norovirus infections occurred throughout the year, however, the majority infections were detected in winter and spring months. This finding is in accordance with previous studies carried out in temperate-climate countries that showed the most norovirus detection in cold seasons [60,61,62,63]. In contrast, in a previous study from our grouper formed between 2017 and 2018, we observed higher norovirus detection rates during the summer/autumn months compared to winter/spring months in symptomatic outpatients [26]. This shift of norovirus seasonal circulation could have been influenced by the unprecedented global disruptions and altered human behaviors during the COVID-19 pandemic. In regard to age groups, we observed the highest prevalence of norovirus infections among children aged 6–24 months, which is consistent with our previous study [26], and other studies from Africa and Asia [42,64,65]. Similar to our data, recent studies performed in China, investigating noroviruses in children with AGE, observed that the viral detection rates were highest in children aged 6–24 months [66,67]. A study performed in northern Brazil with hospitalized children with AGE has shown that the most affected age group was also children between 6 and 24 months old [37]. In our study, the incidence of norovirus was lower in infants aged 0–6 months compared to other age groups, probably due to maternal antibodies that could be a protective factor for children early in their life during the breastfeeding period [68]. In addition, norovirus infections might result in protective immunity against reinfection varying from four to eight years post-infection [21].

A diverse range of the norovirus genotypes was identified throughout the study. We identified five distinct genotypes of norovirus GI, namely GI.2, GI.3, GI.4, GI.5 and GI.7. Notably, GI.4 and GI.3 were the predominant genotypes, which is consistent with findings reported by a global norovirus strain surveillance network, which identified GI.3 as the most prevalent genotype within GI [69]. Previous studies with children under five age who were hospitalized with AGE in Indonesia [70] and Germany [41] also detected GI.4 and GI.3 as the most frequently detected genotypes, respectively. Recently, a study on AGE outbreaks in closed or semi-closed institutions in Catalonia, Spain, between 2017 and 2018, showed that GI.4 (35.7%) and GI.3 (14.3%) were the main genotypes among GI noroviruses [71]. More recently, another study in Spain, has reported the detection of the rare recombinant genotype GI.5[P4], also detected in our study, as the cause of a large foodborne outbreak of AGE occurred in a hotel in 2021 [72].

Thirteen norovirus recombinant genotypes were observed, with nine belonging to norovirus GII, and the most prevalent recombinant genotypes were GII.4[P16], GII.2[P16] and GII.6[P7]. These findings align with previous studies reported in Brazil [24,73] and other countries, including Australia China, India, Malaysian, Greece, Germany, and France [27,39,74,75,76,77,78]. The GII.4 Sydney_2012[P16] strain was first identified in norovirus-associated outbreaks in the United States in November 2015 [60]. Subsequently, this genotype gradually became predominant in outbreaks and sporadic cases of AGE in many countries worldwide [31,69,79]. In Brazil, GII.4[P16] was the most frequently identified genotype in our previous study with patients with AGE symptoms collected between January 2017 and December 2018 [26]. Several reports around the world demonstrating the dominance of GII.4[P16] suggest the increased viral fitness of recombinant genotypes associated with P16 polymerase [80]. A previous study found a high homology of the RdRp gene between the P16 of this novel strain (GII.4[P16]) and GII.2[P16], in addition to the high homology of the VP1 gene of GII.4[P16] and GII.4[P31] [79].

In our study, from September 2020 to June 2021, we detected the recombinant genotype GII.12[P16] as a major strain, prevailing among the genotyped samples during this period. This recombinant strain was first identified in the United States in 2017 [80] and later detected in Canada, where it was associated with epidemic and endemic AGE, becoming the second most predominant strain during the 2018–2019 epidemic season in that country [81]. More recently, a study in China identified the recombinant strain GII.12[P16] in stool and water samples linked to a waterborne AGE outbreak [82]. In Brazil, this recombinant strain was recently detected in bivalve samples collected in the state of Rio de Janeiro [83], and also in stool and ice pop samples related to a foodborne AGE outbreak reported in the South region [84].

Throughout the four-year study period, GII.4 and GII.2 were the most prevalent genotypes detected in Brazil. GII.4 was detected in combination with multiple polymerase types, including GII.P4, GII.P16, and GII.P31, while GII.2 was detected combined only with P16. The recombination of GII.4 with different RdRps may be an important mechanism for its long-term persistence in the population and increase the transmissibility of norovirus. Additionally, a previous study by Parra et al. [15] showed that the GII.4 genotype has the highest rate of evolution within the capsid sequence compared to other genotypes, and continually accumulates mutations in its genome over time. In contrast, “static” genotypes may be genetically fragile, which limits their antigenic diversity and prevalence in the population. According to a recent study in China during the 2019/2020 season, the clinical feature of GII.4-associated AGE was its increased severity, with a longer duration of diarrhea [82]. In Brazil, the prevalence of GII.4 and GII.2 is consistent with our previous study that reported norovirus infections from sporadic cases and outbreaks of AGE from 2017 to 2018 [26], and with the global pattern that has been observed in different countries, such as China, India, Indonesia, Italy, and Germany [41,66,76,83,84].

According to our findings, genotypes carrying a P16 polymerase type were found to be the most prevalent, especially in the first three years, when it was detected at rates over 60% among all norovirus genotyped samples. We detected P16 combined with GII.2, GII.4, and GII.12 capsids, similar to studies that have demonstrated P16 in combination with multiple capsid genotypes, including GII.1, GII.3, GII.13 and GII.10 [60,80]. Due to containing substitutions that enhance RdRp function and virus transmission [85], it is possible that GII.P16 in combination with the fast-evolving GII.4 capsids [15] could have resulted in a highly transmissible virus [86,87,88].

The intergenogroup recombinant GIX.1[GII.P15] was detected in five AGE sporadic cases from the states of Bahia, Rio de Janeiro and Santa Catarina. Globally, GIX.1[GII.P15] is a rare genotype with a low detection rate [89,90]. This genotype was reported during an outbreak investigation from several Chinese provinces during October 2016 and September 2018, where GIX.1[GII.P15] was detected in under 1% of the outbreaks [91]. Unlike GII.4 noroviruses, a recent study suggested a relatively static nature in the evolution of GIX.1[GII.P15] strains, and that the GII.P15 of this genotype might have diverged from the GII.P6 polymerase type [92].

Our study has some limitations. Firstly, the variability in the reporting of AGE cases, and the problems faced by the states in the collection and shipping of stool samples, mostly during the COVID-19 pandemic in Brazil generating surveillance biases. Between April 2020 and June 2021, we observed a sharp decrease in the number of reported AGE and norovirus infections due to intense non-pharmaceutical interventions implemented by Brazilian Federal and State governments to stop the spread of SARS-CoV-2, which changed the patterns of human contact and the epidemiology of infectious agents. Secondly, variability in the reporting and collection of AGE cases among different states further contributed to surveillance biases. Lastly, important and more diverse regions of the norovirus capsid gene, such as the P2 subdomain, were not characterized, and changes in amino acid compositions within the antigenic epitopes were not determined. Future studies are planned to conduct a comprehensive characterization of the P2 subdomain and/or the entire capsid for selected strains in order to follow norovirus evolution over time. 

## 5. Conclusions

In conclusion, our data provide useful information on the molecular epidemiology and genetic diversity of norovirus in Brazil from 2019 to 2022, being the first study to access norovirus epidemiology in Brazil during the COVID-19 pandemic period. Norovirus strains exhibit divergent evolution over time, resulting in a complex genomic diversity that may vary based on factors such as geographic location, age, and other variables [28]. Despite a temporary reduction in norovirus cases during the strict phase of the COVID-19 pandemic, it is crucial to sustain epidemiological surveillance for viral AGE and enhance awareness regarding norovirus outbreaks. Due to the ubiquitous nature of norovirus and considering that the benefits of COVID-19 countermeasures are unsustainable, it was expected that there would be an increase in the incidence of norovirus-associated AGE. Considering that vaccines are in phase III of development, establishing and maintaining an active surveillance network for norovirus typing becomes imperative to monitor the potential emergence of new strains and changes in clinical features and epidemiology. Furthermore, data regarding norovirus strain circulation will play a pivotal role in informing the design of new vaccines.

## Figures and Tables

**Table 1:** Number of norovirus-positive fecal samples identified through laboratory-based surveillance in Brazil from 2019 to 2022.

Region/ State	No. of Fecal Samples–Positive/Tested (%)				
	Total n (%)	2019	2020	2021	2022
**Southeastern**	181/659 (27.4)	72/214 (33.6)	7/61 (11.5)	68/142 (47.8)	34/242 (14.0)
Espírito Santo		35/101	3/13	21/39	5/92
Minas Gerais		16/33	1/18	43/79	22/102
Rio de Janeiro		21/80	3/30	4/24	7/48
**Northeastern**	172/504 (34.1)	71/178 (39.8)	9/106 (8.4)	51/108 (47.2)	39/112 (34.8)
Bahia		42/95	9/105	45/92	13/20
Maranhão		1/1	-	-	-
Paraíba		-	-	-	0/6
Pernambuco		18/62	0/1	7/15	26/81
Sergipe		10/20	-	0/1	0/5
**Southern**	734/1750 (41.9) *	105/340 (30.8)	45/135 (33.3)	455/785 (57.9)	129/490 (26.3)
Rio Grande do Sul		46/181	35/94	351/598	79/315
Santa Catarina		59/159	10/41	104/187	50/175
**Total**	1085/2913 (37.2)	248/732 (33.8)	61/302 (20.1)	574/1035 (55.4) *	202/844 (23.9)

* *p* < 0.05, Chi-square and Fisher’s exact tests.

**Table 2:** Number of tested and norovirus-positive fecal samples by seasons in Brazil during 2019–2022.

Seasons	No. of Fecal Samples—Positive/Tested (%)					*p*-Value ^1^ (Chi-Square Test)
	2019	2020	2021	2022	Total	
**Summer**	80/195	42/138	14/35	24/180	160/548 (29.1)	<0.0001
**Autumn**	95/230	0/31	18/50	74/257	187/568 (32.9)	0.0032
**Winter**	34/137	5/32	186/290	55/240	280/699 (40)	0.8437
**Spring**	39/175	15/103	356/660	50/172	460/1110 (41.4)	-

^1^ *p*-values were calculated between the spring season and each other.

**Table 3:** Number of tested and norovirus-positive fecal samples by age group in Brazil during 2019–2022.

Age Group (Months)	N° of Stool Samples—Positive/Tested (%)					*p*-Value ^1^ (Chi-Square Test)
	2019	2020	2021	2022	Total	
0–6	30/101 (29.7)	3/34 (8.8)	22/52 (42.3)	9/55 (16.3)	64/242 (26.4)	<0.0001
>6–12	45/118 (38.1)	10/36 (27.7)	51/102 (50)	32/86 (37.2)	138/342 (40.3)	0.0470
>12–24	79/173 (45.6)	12/52 (23)	174/246 (70.7)	47/171 (27.4)	312/642 (48.5)	-
>24–60	27/119 (22.6)	9/56 (16)	142/242 (58.6)	48/214 (22.4)	226/631 (35.8)	<0.0001
>60	67/225 (29.7)	27/125 (21.6)	185/393 (47)	67/329 (20.3)	346/1072 (32.2)	<0.0001

^1^ *p*-values were calculated between the age group of >12–24 and each other.

## Data Availability

The data that support the findings of this study are openly available in GenBank database at https://www.ncbi.nlm.nih.gov/genbank/. The datasets generated and analyzed during the current study are available in the GenBank repository under the following accession numbers cited in the Materials and Methods. This study is registered in the Brazilian National System for Genetic Heritage and Associated Traditional Knowledge Management (SisGen, No. A837EB6).
